# Report on the Yellow Fever Epidemic at Wilmington, N. C., in the Autumn of 1862

**Published:** 1864-03

**Authors:** Wm. T. Wragg

**Affiliations:** Surgeon P. A. C. S.


### CONFEDERATE STATES

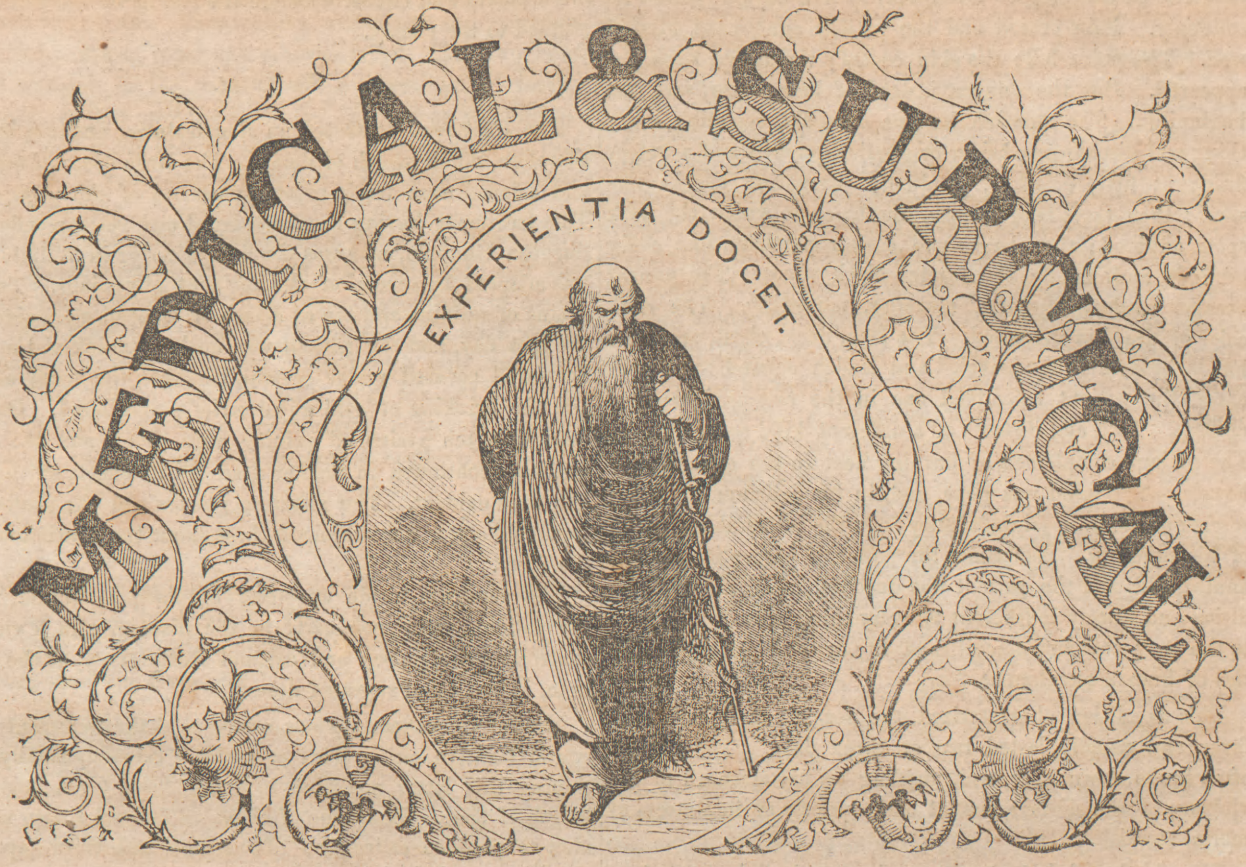

### JOURNAL.

VOL. I. RICHMOND, MARCH, 1864. No. 3.

### ORIGINAL COMMUNICATIONS.

ART. I.—*Report on the Yellow Fever Epidemic at Wilmington, N. C., in the Autumn of* 1862. By WM. T. WRAGG, Surgeon P. A. C. S. (Concluded.)

### *Symptoms.*

The symptoms presented in this epidemic did not differ from those usually described. The attack usually came on suddenly, though occasionally the patient was uncomfortable and unwell for several days before the actual invasion. A chill, in some cases amounting to an actual ague, generally indicated the onset. Pain in the head, back, loins and limbs immediately followed, and very soon there was a feeling of soreness all over the abdomen, from the diaphragm to the pubis. Nausea and vomiting were often attendant upon this stage of the disease, when the contents of the stomach, usually undigested, and then bilious matter, were freely discharged. Fever at once set in, when the heat of the skin was usually intense, though not always dry, for sweating was often profuse, without abating the intenseness of the burning, acrid heat. The pulse was generally frequent, full, hard and burning; sometimes so rapid as to reach 120 or 130 beats in the minute. The headache and pain in the back soon became intense and often unbearable. The face was flushed, and the eyes red and injected. The redness of the eyes sometimes resembled the pink hue so well seen in the white rabbit, sometimes the stupid flush of the drunkard, and often the dry, injected look of inflammation from long exposure to cold, high wind. The flush of the face, though at first florid and red, with an active capillary circulation, quickly returning into the vessels after having been forced out by pressure with the finger, was, on the contrary, in other cases dull, dark and dusky, with a sluggish circulation, slowly returning into the vessels when pressed away with the finger, and giving the patient the appearance of having been thoroughly smoked.

In the first twenty-four or thirty-six hours, these symptoms steadily increased The headache and pain in the back became unbearable. The heat of the skin was often intense. Sighing, moaning and tossing about were incessant. Delirium and wakefulness marked the nights Thirst and a burning, inward heat tormented the patient. Oppression and a fullness at the chest, as if there was something pressing upwards, induced him to believe that he would be relieved if he could only discharge the load by belching or vomiting. Weakness, faintness, dizziness and dimness of vision made the slightest attempt to rise or move difficult.

This formed the first stadium of the fever. Death seldom occurred in this stage. After four or five days, it passed off suddenly. Pain ceased and the patient announced himself well. The pulse fell; the skin cooled; the active flush disappeared. But the danger, instead of being over, was just beginning. The stomach became irritable, rejecting every thing and discharging larger or smaller quantities of a watery fluid; at first, colorless, gradually presenting specks like snuff; then threads of mucus, like hairs, to which the dark spots adhered; then larger and darker particles, like coffee grounds, and, finally, the fearful black vomit. The patient became restless—sighing, moaning, muttering. The dark color of the skin increased. The face, neck, chest, arms and hands became cold, shriveled and leaden colored. In some cases, the patient resembled one in the collapsed stage of cholera. Pain and distress of a different kind soon came on. The patient complained of burning heat in the stomach and bowels. The mind was gone, and, in his delirium, he would scream or groan in frightful fury from the intolerable anguish of his suffering. Sometimes the power of perception seemed annihilated, and the patient was unconscious of his own condition and utterly indifferent to every thing around him, even to the most ordinary amenities of life or the most engrossing ties of human attachment.

This condition characterized the second stadium of the fever and usually lasted two or three days. The patient often broke down here, and, yielding up his strength to the violence of the disease, passed away quietly or in a storm of mortal anguish.

The third and last stage was characterized by a continuance of many of the same symptoms and by the addition of others. The black vomit increased in quantity and changed to a still darker hue. It became thicker, and was often so putrid and offensive as to test the endurance of the most experienced nurses. The stomach would retain nothing; even a teaspoonful of anything producing the most excruciatingly painful retching. The capillary circulation seemed almost arrested. The urinary secretion was often totally abolished, and this was a symptom after which no patient ever recovered. Nothing was so positively fatal as this symptom. The eyes and skin often became jaundiced in a few hours, and, in some cases, so intensely as to render the patient unrecognizable. Hemorrhages, in some cases, occurred from the gums, tongue, nose, bowels and uterus. The odor from the patient's body was offensive in the extreme. The tongue and mouth were covered with putrefying blood, and the breath offensive beyond description. About this period of the disease, though sometimes at an earlier stage, hiccough was often added to the dreadful array of symptoms that racked the tortured patient. Coming on in paroxysms and lasting a longer or shorter time, it would wear out the small remnant of strength still left him, appearing to lacerate and root up the last tendrils through which the wasted sap of life was flowing.

From this last stage, recoveries, though rare, were sometimes met with. When they occurred, the convalescence was slow and relapses not unfrequent, as the consequence of imprudent exposure. When death occurred, the body was often putrid and offensive before the citadel of life had fairly yielded to the enemy.

*Treatment.*

The treatment presented no new features, and needs not, therefore, much length of detail. The large doses of quinine and calomel were freely given by several of the physicians. This treatment having been employed and recommended by Surgeon Choppin, who was the first to arrive in Wilmington, was adopted by several of the resident physicians. Twenty grains of quinine and ten or fifteen of calomel were given on the first day, and the patient was made to drink warm teas, keep well covered under several blankets, the room closed, and the drinking of cold water strictly prohibited. On the next day, ten grains of quinine were given and the same medicine continued in smaller doses till its effects on the system were evident. The sweating process and the abstinence from cold water were enjoined throughout the continuance of the fever.

The veratrum viride was used by Surgeon White, when the patients were seen early in the disease, with a view of controlling the pulse; and calomel freely given, at the same time, in small and repeated doses.

Most of the other surgeons commenced the treatment by a gentle laxative, either with or without calomel, usually continuing this latter medicine in small doses for a moderate length of time. But none of them, as far as I know, with a view to produce salivation. The second and third day were usually passed in administering gentle diaphoretics, often combined with small quantities of quinine, and, at the same time, prescribing for any of the symptoms which seemed most urgent. The use of blisters over the abdomen, extending from the diaphragm to the pubis, was general. Hot mustard pediluvia, with cold applications to the head, were much employed during the fever stage. Hot mustard poultices to the chest, back or loins, when these regions were painful, gave great relief. Croton oil liniment was employed on the chest when the oppression and weight, or the sensation as if relief would follow vomiting or belching, were distressing.

In my own practice, I found the free use of the cold douche upon the head not only relieved the intense headache and calmed the restlessness, but quieted the pulse, causing it to diminish in frequency, hardness and fullness. It was performed by turning the patient's head and shoulders over an empty tub at the side of the bed, and pouring a full stream of cold water from a pitcher over the whole head, taking care to guard the ears and eyes. This was repeated at short intervals and as often as the patient called for it.

Throughout the disease, light nutritive matter was allowed, and, as soon as the febrile symptoms permitted, the use of stimulants was added to that of nutriment.

To allay irritability of the stomach, a great number of remedies were suggested and tried, but, in the nausea and vomiting following the fever stage and preceding or ushering in black vomit and the other formidable symptoms of the apyrexia, they were of little avail. Acid, alkaline and neutral mixtures were used. Carminatives, stimulants and narcotics were tried. Musk, ether and creasote failed. The functions of the stomach, in the worst of these cases, seemed totally destroyed, so that nothing could be digested or assimilated. Its peristaltic action seemed reversed, so that every thing that was put into it was instantly returned. The poison of disease had done its duty.

Sometimes the work of destruction was not so thoroughly done. Small portions of ingesta were retained, and the stomach, like the other organs, seemed gradually to disenthral itself from the power of the poison. In such cases, champagne in small quantities, brandy with milk and lime water, arrow root and brandy, jelly, beef tea, and so on to other and more substantial nourishment, were retained and assimilated, and the dawn of hope would brighten up for the rescued victim.

*Mortality.*

It only remains for me now to give an idea of the extent of the disease and of its ravages. The "Wilmington Journal," of the 17th of November, gives the following statement of the progress of the fever, viz:

| | | |
|---|---|---|
| Up to September 19th............... | 8 cases, | 6 deaths. |
| Week ending September 26th..... | 26 " | 9 " |
| " " October 3d........... | 267 " | 82 " |
| " " " 10th.......... | 395 " | 40 " |
| " " " 17th......... | 431 " | 102 " |
| " " " 24th......... | 194 " | 111 " |
| " " November 1st........ | 116 " | 40 " |
| " " " 8th........ | 47 " | 30 " |
| " " " 15th........ | 21 " | 21 " |
| Up to November 17th............... | 2 " | 5 " |
| Total, | 1,507 cases. | 446 deaths. |

This exhibits a very great mortality. It is about one in three and a third; thus showing the virulence of the disease. About this percentage is usually met with in hospital practice, where the patients generally come in late and have fewer chances of life than in domestic practice, where they are seen early. Much of the unsuccessful results of private practice here must be attributed to the unfavorable circumstances attending the sick; such as want of good nursing, of articles required for the nourishment of the patients, bad cooking, and, by no means the least of all, to the depressing effects of panic.

The population of Wilmington during the epidemic was variously estimated by competent observers at from 4,000 to 6,000. My own impression is that 5,000 would not be far from the truth. That would give about one case of fever out of every three and a quarter inhabitants, and one death out of about every eleven. But if we remember that, of the population remaining in Wilmington, at least one-half were negroes, and that negroes are much less liable to the disease than whites, is will appear that the ratio of sickness and mortality among this latter class are much greater than what is stated.

